# Beyond mineral metabolism, the bright immunomodulatory effect of vitamin D in renal disease

**Published:** 2012-07-01

**Authors:** Azar Baradaran

**Affiliations:** Department of Pathology, Isfahan University of Medical Sciences, Isfahan, Iran

**Keywords:** Vitamin D, 25-hydroxyvitamin D, Kidney

**Implication for health policy/practice/research/medical education:**

Base on the potential immunomodulatory effect of vitamin D, the association of vitamin D status and renal disease and the effect of vitamin D supplementation in kidney disease patients, further investigations suggests.

Calcitriol is the hormonal form of vitamin D and is formed by two hydroxylation steps: a hepatic 25-hydroxylation of vitamin D and a subsequent kidney 1 alpha-hydroxylation (1-3). The active metabolite of vitamin D3, 1, 25-dihydroxyvitamin D3, is a hormone which principally regulates calcium and bone metabolism. Deficiency of vitamin D is public globally. While the integrity of the vitamin D is crucial for human health, nutritional vitamin D deficiency in otherwise healthy persons, associates with a higher risk of mortality for all causes, in spite of normal serum calcitriol (2-4). Besides of its classical actions, calcitriol applies its pleiotropic properties in a wide variety of target organs and cell types, often in an autocrine/paracrine manner. These biological actions of calcitriol have suggested a multitude of potential therapeutic determinations for the vitamin D hormone in the therapy of hyperproliferative disorders (e.g. psoriasis and cancer) and immune dysfunction (1-4). Deficiency of vitamin D links with an early onset of a variety of disorders such as high blood pressure, insulin resistance, proteinuria and immune abnormalities that enhance the tendency for viral and bacterial infections, and multiple organ injury due to systemic inflammation causing atherosclerosis, impaired DNA-damage responses and renal lesions too. It is noteworthy to remember that the physiological action of 1, 25-dihydroxyvitamin D3 is mediated by the receptor of vitamin D (3-5). Vitamin D receptor (VDR) is discovered in various organs and cells including small intestine, bone and kidney. Furthermore to the regulation of calcium metabolism, calcitriol is involved in various biological reactions such as differentiation induction, anti-proliferative effect, immunomodulatory effect, and regulation of cytokine and parathyroid hormone secretion, while, all of these disorders obviously increase in chronic renal failure because the kidney is essential to maintain serum levels of 1, 25-dihydroxyvitamin D3, the most potent endogenous endocrine activator of the VDR, and also of 25-hydroxyvitamin D, for local rather than systemic VDR activation. Recent data shows, local production of 1, 25-dihydroxyvitamin D (1,25(OH)(2)D) regulated by the CYP27B1 enzyme in monocytes contributes to the immunomodulatory effects of vitamin D and uremia suppresses kidney CYP27B1 (1-6). In fact monocytic baseline CYP27B1 expression is increased in uremia, probably reflecting the micro-inflammatory condition. Immune signal-induced CYP27B1 expression, conversely, is impaired in uremic conditions. On the other hand, elevated fibroblast growth factor 23 (FGF23) levels, may account, at least partly, for the dysregulation of monocytic CYP27B1 in uremia and, as such, may contribute to the high cardiovascular and infectious disease and immune system disorder in chronic renal failure (5-10). Principally FGF23 is an “endocrine” FGF conducting in the kidney as a phosphaturic hormone and also a suppressor of active vitamin D, by an inhibition of the 1α hydroxylase and activation of the 24 hydroxylase (6-10). To find the vitamin D status of Chinese kidney transplant recipients, Ma *et al.* conducted a cross-sectional study to measure the level of 25-hydroxyvitamin D in 94 Chinese kidney transplant patients with stable allograft function. They found vitamin D deficiency and insufficiency were found in 43.6% and 54.2% of patients, respectively. The level of 25-hydroxyvitamin D was lower in renal transplant recipients compared with healthy persons matched for age and sex. Interestingly, they found, patients with a history of acute rejection had lower levels of 25-hydroxyvitamin D (11). They observed that vitamin D deficiency is prevalent among Chinese renal transplant recipients (11). Base on the potential immunomodulatory effect of vitamin D, the association of vitamin D status and renal disease and the effect of vitamin D supplementation in kidney transplant patients, further investigations suggests.

## Author’s contribution

AB is the single author of the manuscript.

## Conflict of interests

The author declared no competing interests.

## Ethical considerations

Ethical issues (including plagiarism, data fabrication, double publication) have been completely observed by the author.

## Funding/Support

None.
